# Invasive Fungal Infections: The Early Killer after Liver Transplantation

**DOI:** 10.3390/jof9060655

**Published:** 2023-06-12

**Authors:** Robert Breitkopf, Benedikt Treml, Zoran Bukumiric, Nicole Innerhofer, Margot Fodor, Sasa Rajsic

**Affiliations:** 1 Department of Anesthesia and Intensive Care Medicine, Medical University Innsbruck, 6020 Innsbruck, Austria; 2 Institute of Medical Statistics and Informatics, Faculty of Medicine, University of Belgrade, 11000 Belgrade, Serbia; 3 Department of Visceral, Transplantation and Thoracic Surgery, Medical University of Innsbruck, 6020 Innsbruck, Austria

**Keywords:** liver transplantation, survival, mortality, adverse events, invasive fungal infections, transplantation, liver

## Abstract

Background: Liver transplantation is a standard of care and a life-saving procedure for end-stage liver diseases and certain malignancies. The evidence on predictors and risk factors for poor outcomes is lacking. Therefore, we aimed to identify potential risk factors for mortality and to report on overall 90-day mortality after orthotopic liver transplantation (OLT), especially focusing on the role of fungal infections. Methods: We retrospectively reviewed medical charts of all patients undergoing OLT at a tertiary university center in Europe. Results: From 299 patients, 214 adult patients who received a first-time OLT were included. The OLT indication was mainly due to tumors (42%, 89/214) and cirrhosis (32%, 68/214), including acute liver failure in 4.7% (10/214) of patients. In total, 8% (17/214) of patients died within the first three months, with a median time to death of 15 (1–80) days. Despite a targeted antimycotic prophylaxis using echinocandins, invasive fungal infections occurred in 12% (26/214) of the patients. In the multivariate analysis, patients with invasive fungal infections had an almost five times higher chance of death (HR 4.6, 95% CI 1.1–18.8; *p* = 0.032). Conclusions: Short-term mortality after OLT is mainly determined by infectious and procedural complications. Fungal breakthrough infections are becoming a growing concern. Procedural, host, and fungal factors can contribute to a failure of prophylaxis. Finally, invasive fungal infections may be a potentially modifiable risk factor, but the ideal perioperative antimycotic prophylaxis has yet to be determined.

## 1. Introduction

Since the first human orthotopic liver transplantation (OLT) in 1963, advances in surgical techniques and perioperative management, organ allocation and preservation, immunosuppression, and the management of postoperative complications have transformed the experimental procedure into an established standard of care treatment for end-stage liver disease, certain malignancies, and acute liver conditions with life-threatening hepatic dysfunction [1,2]. 

However, the clinical field of OLT has significantly changed during the last decades. Due to the persistent shortage of organs and increasing mortality of patients on the waiting list, the selection criteria for organ acceptance, but also the list of indications, as well as the comorbidity and frailty of the patients, have evolved [3]. Improved perioperative and postoperative management of OLT recipients has reduced the number of absolute contraindications. Therefore, potential recipients are beeing older, and more likely to have diabetes and to be obese, with increasing incidence of portal vein thrombosis in an allocation system where the sickest candidates are prioritized and exclusion criteria are not specified [4,5]. Moreover, human immunodeficiency virus (HIV) is no longer an absolute contraindication [6]. 

Using extended criteria donation (ECD) and donation-after-circulatory-death (DCD) grafts has led to the expansion of the potential donor pool, but also the deterioration of the donated organs’ quality over the years [7,8]. Surgically demanding living donor liver transplantations are becoming more and more established in the clinical routine [9]. The development of direct-acting antiviral drugs reduces the indication of hepatitis C virus-related cirrhosis, whereas malignancies (e.g., cholangiocarcinoma, metastatic neuroendocrine tumors, etc.) or nonalcoholic steatohepatitis are becoming feasible indications [10,11,12,13].

Although long-term outcomes have greatly improved over time, the early postoperative period remains critical. Previously published overall in-hospital mortality rates vary between 6.3% and 8.4%, and can be up to 13.6% for deceased-donor liver transplantation [14,15,16]. The European Liver Transplant Registry reported that 46% of deaths and 65% of re-transplantations occur within the first half year after OLT, with almost 50% of graft failures and one quarter of deaths occurring within the first month [11].

Technical factors and complications (e.g., hemorrhage, vascular and biliary complications, hepatic infarction, etc.), and cardiovascular diseases, cerebrovascular accidents, and respiratory failure have often been described as predominant causes of death in the early postoperative course, whereas infections and graft dysfunction (e.g., primary non-function, acute rejection, etc.) have been the main determinants of short-term mortality. However, little is known about the early postoperative mortality or the role of fungal infections in the current era [16,17,18,19].

The incidence of IFI in liver transplant recipients ranges from 4 to 40% and increases with the time after OLT. The overall rate of 1.8% after one year rises up to 2.9% after 5 years and 5% after 10 years. *Candida* spp. are the most common fungal pathogens (68–93%), mostly based on endogenous infection arising from preexisting colonization. Surgical site infections, such as peritonitis and abdominal abscesses, are the most common forms of intra-abdominal candidiasis, followed by biliary tract infections, which can complicate treatment due to the decreased tissue penetration of antifungal drugs. Invasive *Candida* infections typically occur in the early postoperative period after transplantation, with approximately 34% and 46% of cases occurring in the first month and within three months, respectively. Invasive *Aspergillus* infection occurs in 1% to 9% of patients after liver transplantation, and mostly affects the airways and/or sinuses. In comparison to *Candida* infections, *Aspergillus* tends to occur in the later postoperative period. Although previous epidemiological studies have reported an early onset of invasive aspergillosis within 17 days after transplantation, more recent trials suggest a rather delayed infection (more than 100 days), with disseminated aspergillosis being common in liver transplant [20].

Given the above, the aim of this study is to investigate the overall 90-day and one-year mortality, especially focusing on procedural and infectious factors. Moreover, we provide a summary and comparison of the demographic and clinical characteristics of a mixed population of OLT recipients, while focusing on perioperative predictors and risk factors for short-term mortality.

## 2. Materials and Methods

### 2.1. Patient Selection

All OLT recipients between January 2017 and December 2020 at the Medical University Innsbruck, Austria, were assessed for eligibility. We included patients older than 18 years who received OLT. Exclusion criteria were combined liver−kidney transplantation, living donor liver recipients, re-transplantations (after more than 90 days after the initial transplantation), and multivisceral transplantations. In the case of liver re-transplantation within 90 days after the first organ transplantation, only data from the first operation were analyzed.

This work was prepared and revised according to the ‘strengthening the reporting of observational studies in epidemiology’ (STROBE) statement checklist of items (Supplementary Table S1). 

### 2.2. Data Collection and Study Endpoints

The primary endpoint of our work was 90-day mortality after OLT. The secondary endpoints included the identification of risk factors for mortality after OLT, especially focusing on infections (e.g., invasive fungal infections, other opportunistic or hospital-acquired infections, etc.), as well as procedural factors (e.g., graft failure, immunologic, vascular, and biliary complications, postoperative hemorrhage, acute kidney injury, etc.), and one-year mortality. 

We reviewed the electronic medical records of all OLT recipients. Data obtained included (1) sociodemographic data; (2) underlying indications for OLT, the severity of disease (as measured by MELD score and SAPS III score), and the comorbidities (Charlson Comorbidity Index); (3) basic information on surgical technique, organ donation, and preservation; (4) data on immunosuppression and antimicrobial prophylaxis; (5) postoperative complications, ICU- and hospital stays; and finally (6) data on cause and date of death from the clinical or post-mortem examinations. The information on the date of death was recorded, and the mortality in different periods was calculated.

### 2.3. Surgical Technique and Outcomes Definition

A standard OLT was defined as the deceased donor transplantation of a standard criteria donor organ after static cold storage. The recipient hepatectomy was performed by retrohepatic caval resection without a veno-venous bypass, and the biliary anastomosis by duct-to-duct reconstruction. The technique deviations were additionally recorded (e.g., extended criteria donation, split liver donation, donation after circulatory determination of death, an inferior vena cava preservation by “piggyback” technique, the use of a veno-venous bypass, or a Roux-en-y choledochojejunostomy).

Normothermic machine perfusion (NMP) has been used since February 2018 at our center and has been implemented in a daily routine for the selected indications. The selected donor-related indications included extended criteria donation, especially if prolonged ischemia times are expected; recipient-related indications included high-risk patients or surgically complex recipients; and logistic-related indications were applied in case of limited resources (e.g., overlap with other urgent interventions, parallel organ transplantations, etc.).

#### Outcome Definitions

The diagnosis of a “proven” invasive fungal infection (IFI) was based on the definitions of the European Organization for Research and Treatment of Cancer/Invasive Fungal Infections Cooperative Group and the National Institute of Allergy and Infectious Diseases Mycoses Study Group (EORTC/MSG) Consensus Group [21]. Upon the recommendations of the EORTC/MSGERC ICU Working Group, a “probable” disease was diagnosed depending on the level of probability for invasive infection diagnosis in a critical care setting [22]. Breakthrough infections were defined as by the Mycoses Study Group Education and Research Consortium (MSG-ERC) and the European Confederation of Medical Mycology (ECMM) [23].

The diagnosis of an invasive pulmonary aspergillosis (IA) was made upon a combination of the following criteria: (1) presence of clinical and host factor criteria, (2) at least one clinical/radiographic finding suggestive of IA (e.g., computed tomography, bronchoscopy), and (3) at least one mycological evidence of *Aspergillus* spp., (e.g., positive culture from a normally sterile site such as bronchoalveolar lavage fluid—BAL) with subsequent molecular testing for defining the specific species, Galactomannan antigen detection via enzyme immunoassay in BAL and/or serum, or polymerase chain reaction performed on serum, plasma, whole blood, or BAL fluid. 

We defined early mortality as death occurring within the first 30 days after transplantation, and short-term mortality as death occurring within the first 90 days. Invasive-fungal-infections-attributable mortality is defined as all-cause mortality, excluding patients who died due to the underlying disease process. Death was attributed to IFI if the clinical course of infection was refractory to treatment at the time of death (i.e., stable disease or disease progression), if the patients died as a result of an acute event at one of the sites of infection or otherwise unexplainable cause of death in the course of established infection, and if patients have died as a result of the toxicity of antifungal therapy.

### 2.4. Immunosuppressive Regimen and Prophylaxis 

The immunosuppressive therapy in our OLT recipients was based on the local standard algorithm for ABO-compatible transplantation. This algorithm comprised an intraoperative 500 mg methylprednisolone bolus with subsequent tapering over five weeks, in a triple combination with an antimetabolite and a calcineurin inhibitor. 

Preoperative selective digestive decontamination (oral amphotericin B and nonabsorbable antibiotics) was used in the elective OLT recipients. 

All patients received antibiotic prophylaxis with piperacillin/tazobactam (or levofloxacin in case of allergies) for five days. In high-risk constellations based on donor/recipient serostatus (i.e., seronegative recipients from seropositive donors), antiviral prophylaxis with valganciclovir was carried out (duration of 3–6 months). The antifungal prophylaxis using an echinocandin (micafungin, anidulafungin, for duration of 7–14 days) was reserved for high-risk OLT recipients. The high-risk recipients were defined as those having a combination of ≥2 predefined perioperative risk factors (e.g., retransplantation, pre-existing renal dysfunction, pre-colonization, choledochojejuno-stomy, massive transfusion, increased operating time, etc.) [24,25]. The prophylaxis was carried out for 7–14 days in case of an uncomplicated postoperative course. We used fluconazole, voriconazole or liposomal amphotericin B in cases of a preexisting fungal colonization with echinocandin-resistant *Candida* spp. or *Aspergillus* spp. 

Patients with liver cirrhosis are especially susceptible to infections (e.g., due to abnormalities in humoral and cell-mediated immunity and the occurrence of bacterial translocation from the gut), and at a particular risk of IFI (frequent exposure to broad-spectrum antibiotics, invasive procedures, and prolonged hospital stays, etc.), often associated with delayed diagnosis and high mortality. Although donor and/or recipient colonization is associated with an increased risk of infection, carrier status does not currently constitute a contraindication to transplantation [26], but rather requires precautions for contact isolation and strict adherence to a hygiene regimen. 

In case of a suspicion of acute cellular rejection, depending on the clinical condition of the patient, a timely biopsy was performed. Histological examinations of biopsy specimens were performed by an expert pathologist evaluating portal inflammation, bile duct inflammation damage and venous endothelial inflammation. All acute cellular rejections were classified using the Banff Rejection Activity Index, and further treatment was based on histologic evidence. Mild rejections (i.e., rejection activity index ≤ 4) were treated by optimizing the baseline immunosuppressive regimen, whereas biopsy-proven moderate-to-severe acute rejections (i.e., RAI ≥ 5) received high-dose glucocorticoids (e.g., methylprednisolone 500–1000 mg for one to three days) followed by a glucocorticoid taper, in addition to optimizing the maintenance immunosuppression regimen. At our institution, antithymocyte globulin (ATG) is the preferred regimen for the rare cases of biopsy-proven, glucocorticoid-refractory acute rejection (1.5 mg/kg daily, intravenously, 5–7 days. 

Finally, we performed weekly routine microbiological screening for the active surveillance of healthcare-associated infections (HAI), including fungal surveillance cultures from swabs of the throat, perineum, and urine cultures. HAI were defined using the ECDC criteria, and antimicrobial susceptibility testing was conducted using the European Committee on Antimicrobial Susceptibility Testing standards [27,28,29]. Positive samples taken via drains more than 24 h in situ, as well as from respiratory secretions, stool, skin, wound sites, and an asymptomatic candiduria, were interpreted as colonization and were not treated. If feasible, the management of colonization was directed at the elimination of predisposing factors. Fungal surveillance cultures were used to specify any colonization and provide targeted therapy in the case of infection signs.

### 2.5. Statistical Analyses

All statistical analyses were performed using the SPSS (Version 22.0. Released 2013, Armonk, NY, USA: IBM Corp.). A significance level of 0.05 was applied, and statistical assessments were two-sided. Depending on the normality of the data distribution and the type of variables, results are presented as frequency (percent), mean with standard deviation, and median (range, minimum–maximum). We used the independent samples t-test for parametric data, and the Mann–Whitney U test for ordinal and numeric data with non-normal distribution. Chi-square and Fisher’s exact tests were used to test differences between the nominal data (frequencies). Potential risk factors for mortality were analyzed in a univariate Cox proportional hazards model. Covariates with a significance level of *p* < 0.1 were included in a multivariate model. A Kaplan–Meier estimate was used to analyze the time to death.

## 3. Results

### 3.1. Study Population and Baseline Characteristics

**Figure 1:**
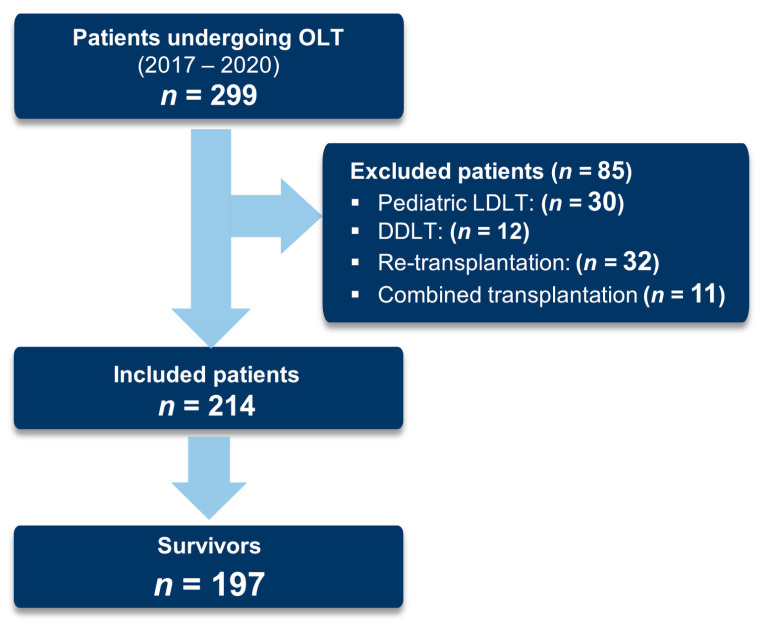
Flowchart of patient selection. Abbreviations: OLT: orthotopic liver transplantation, LDLT: living donor liver transplantations, DDLT: deceased-donor liver transplantation.

Over a period of four years, 299 patients underwent OLT, 214 met our inclusion criteria, and 197 survived the first 90 days after transplantation; see Figure 1. 

The mean age of our patient population was 57 ± 11 years, with a mean SAPS III score of 45 ± 9 and a median Charlson comorbidity index of 4 (0–12). The patients’ demographic and clinical characteristics are presented in Table 1.

In 42% (89/214) of cases the OLT was indicated due to tumors, and in 32% (68/214) due to cirrhosis, mainly related to alcohol abuse (24%, 51/214). Acute liver failure led to high urgency transplantation in 5% (10/214) of patients; see Table 1. The average surgery time was 355 (173–783) minutes, with whole liver and duct-to-duct biliary anastomosis as a dominating surgical approach; see Table 2.

The median duration of the initial postoperative ICU stay was 5 (1–117) days, and in 44% (94/214) of cases it was complicated due to acute kidney injury or the need for re-operation (35%, 74/214). Moreover, re-operation was in 9% (20/214) of cases indicated by a bile leak, in 17% (31/214) by hemorrhage, and in 3% (6/214) there was a need for re-transplantation; see Table 3.

Acute cellular rejection occurred in 5% of the patients. More than half of the diagnoses were based on histopathological examinations, each showing only mild to moderate rejection activity, which could be successfully treated with methylprednisolone and optimization of immunosuppression.

### 3.2. Postoperative Complications

The postoperative course of patients who died within 90 days was mostly characterized by multifactorial adverse events, in average six per patient; see Table 4.

### 3.3. Mortality

We observed 90-day and one-year mortality of 8% (17/214) and 14% (29/214), respectively. Among the seventeen patients who died within the first 90 days, four died within the first week after transplantation, seven in the period of 8–30 days (early mortality), and six in the period from 31 to 90 days after OLT. Infections (5%, 4/214) and procedural complications (2%, 4/214) were mainly responsible for the early deaths (<30 days), followed by intracerebral bleeding (1%, 2/214) and myocardial infarction (1%, 2/214). All patients who died within the first 90 days died within the same hospital admission as OLT.

#### 3.3.1. One-Year Mortality

Major causes of death within the first year after OLT were attributed to infections (8%, 18/214) and procedural complications (2%, 4/214).

In the period from 90 days to one year, twelve (6%, 12/214) additional patients died, eight due to sepsis (67%, 8/12), two patients due to malignancies (post-transplant lymphoproliferative disorder; 17%, 2/12), one patient from acute respiratory distress syndrome caused by Coronavirus disease (COVID-19), and another one due to graft failure as a consequence of an early hepatic artery thrombosis (at the 22nd postoperative day). The information on procedure-attributed mortality is provided in the Supplementary Materials.

#### 3.3.2. Infection-Attributed Mortality

Fungal (39%, 7/18) and respiratory infections (17%, 3/18 hospital-acquired—HAP or ventilator-associated pneumonia—VAP) were the main cause of death within the first 30 days. Surgical site infections (33%, 6/18) and soft tissue infections (necrotizing skin and soft tissue infection in one patient), as well as urinary tract infection in another one patient, were responsible for mortality within the first 90 days. Only one death was caused by VAP after 90 days, as the postoperative course was complicated by central pontine myelinolysis and posterior reversible encephalopathy syndrome.

Bacterial infections were causal in 61% (11/18) of deceased patients, and in an additional two patients they were a contributory death factor. More than half of the lethal bacterial infections were caused by multi-drug-resistant organisms—MDR (Enterobacteriaceae (*Klebsiella* spp.) and *Pseudomonas aeruginosa*, linezolid-(LRE) and vancomycin-resistant *Enterococcus faecium* (VRE), linezolid-resistant *Staphylococcus epidermidis* (LRSE)). Most of the MDR infections (71%, 5/7) occurred more than three months after the OLT. From the remaining two patients (29%, 2/7), one was pre-hospitalized due to hepatic decompensation with hepatorenal kidney failure more than two weeks prior to OLT, and the other died from open tuberculosis, in the clinical course of a *Clostridioides difficile* infection.

Within the lethal IFI category, five patients died from an IA after a median of 43 (15–110) days, whereas one died from an invasive intra-abdominal candidiasis (IAC) without candidemia (*C. krusei*, 48th day), and another one due to a donor-derived IFI caused by *Geotrichum capitatum* 178 days after OLT. In 80% of all IA cases in deceased patients, an accompanying co-infection was found; see Table 5. Sixty percent of the IA infections were confirmed post-mortem by histopathologic examination.

Altogether, nine patients suffered from IC, and four patients had a contributary but not primarily fatal CMV infection. Five further patients were diagnosed with an IC (three as IAC, one as a catheter-related isolated candidemia, and another one as donor-derived infection).

**Figure 2:**
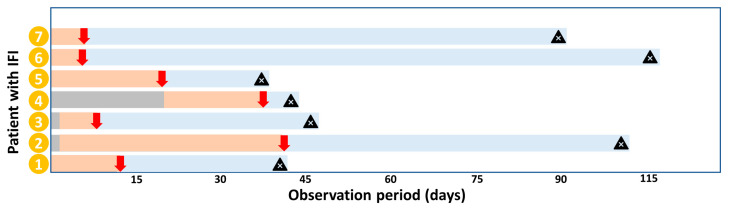
Flow of patients with IFI. Legend: Beginning of prophylaxis (red line), no prophylaxis (gray line), treatment (blue line), moment of IFI diagnosis (red arrow), death (black triangle). Abbreviations: IFI: invasive fungal infection.

Seven of the IC infections occurred as a breakthrough infection during ongoing echinocandin prophylaxis for a median duration of 12 (8–40) days; see Figure 2. In one case, we detected intrinsically echinocandin-resistant *C. parapsilosis* and *C. orthopsilosis* strains (one of which also developed secondary fluconazole resistance) and identified a *C. glabrata* strain, which developed secondary echinocandin resistance after prolonged drug exposure in the clinical context of a tertiary peritonitis.

### 3.4. Risk Factors for Early Postoperative Mortality

The univariate analysis of risk factors for 90-day mortality identified an acute liver failure, the occurrence of a graft-versus-host-disease, postoperative PVT, reoperation, acute kidney injury, CMV viremia, sepsis, and IFI as factors with increased mortality risk; see Supplementary Table S3.

**Figure 3:**
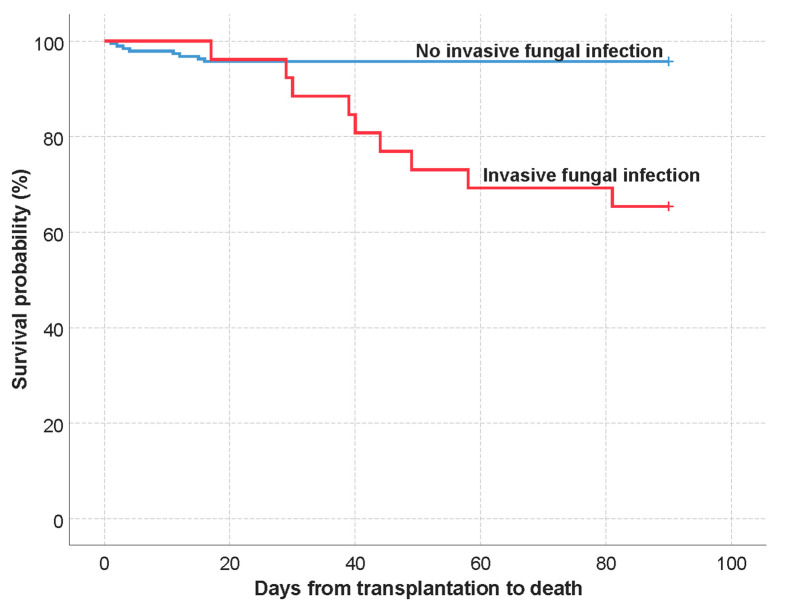
Kaplan–Meier mean survival estimate: analysis of the invasive fungal infection presence (90-day mortality).

Finally, the multivariate analysis identified IFI as the only independent risk factor for early postoperative mortality (HR 4.6, 95% CI 1.1–18.8; *p* = 0.032); see Table 6. The Kaplan–Meier mean survival estimate comparing the survival probability in case of IFI presence is presented on Figure 3. Furthermore, in a second model including sepsis instead of IFI (including sepsis due to IFI), the sepsis (HR 46.7, 95% CI 7.9–277.7; *p* < 0.001) and graft-versus-host disease (HR 26.3, 95% CI 1.5–466.7; *p* = 0.026) were associated with an increased risk of mortality; see Supplementary Table S4.

Age, male sex, BMI, underlying disease, MELD-score, intraoperative blood loss, and co-morbidity expressed by the Charlson Comorbidity Index were not associated with an elevated risk for early postoperative mortality.

## 4. Discussion

Liver transplantation is a potentially life-saving treatment for end-stage liver disease and certain malignancies. Throughout the last decades long-term outcomes have markedly improved, but in-hospital mortality for deceased liver donations is still high (10%) [14,15,16]. Studies examining the causes and course of early postoperative death are sparse, although this is the most critical period after the OLT [11,19,30,31]. Our work is one of the first to examine patient, procedural, and infectious factors in an integrative manner over the course of the postoperative period.

We identified the often-underestimated IFI as a potentially modifiable and independent risk factor for short-term mortality, in addition to procedural (mainly vascular) complications and mostly unavoidable hospital-acquired infections (pneumonia, followed by complex surgical site infections, often with MDR pathogens). Furthermore, we observed an increase in the proportion of infection-related deaths of up to 62% within the first few years. Finally, patient-related comorbidities (e.g., CVA, CVD, etc.) caused less than two percent of postoperative deaths.

### 4.1. Mortality

We found a slightly lower 90-day mortality rate compared to the literature, which may be a consequence of excluding patients with re-transplantations from our work [32,33,34].

In our study, the two major causes of short-term mortality were infections (59%) and procedural complications (18%), which was consistent with recently published works [16,32]. In the censored one-year mortality (excluding the first 90 days), infections not only remained the main cause of death, but increased up to almost 80%, also in line with the literature [35].

#### Infection-Attributed Mortality

Due to complex surgical procedures, including the penetration of the hepatobiliary system, OLT recipients are especially prone to infections [36]. The type of infection and causative pathogen varies depending on the time of onset [37,38]. In our study, half of the early fatal infections were due to hospital-acquired or opportunistic (mainly fungal) infections. In addition to SSI and healthcare-associated pneumonia as the two main infection sites, complicated urinary tract infection and severe *Clostridium difficile* colitis were associated with increased perioperative mortality. All cases of the fatal early SSI were susceptible to the perioperatively used antibiotic prophylaxis, confirming the right choice of agent. However, its duration may be one of the risk factors for the rather high incidence of IFI.

Recent studies have described the 90-day IFI rate after OLT as between 1.4% and 11.1% [39,40,41,42,43,44,45,46,47,48]. We found an IFI rate of 12% within the 90 days, with a case-fatality rate of 35% despite a consequent targeted antimycotic echinocandin prophylaxis in high-risk recipients. Interestingly, two IFI cases resulted from donor-derived infections.

*Candida* was the predominant pathogen in our study, causing 81% of the IFI. Two-thirds of postoperative *Candida* infections (mainly non-albicans species) were located in abdominal surgical sites, where echinocandins exhibit poor penetration (peritoneal and biliary fluid). The alarming rise of non-albicans *Candida* species exacerbates concerns about the prophylactic use of echinocandins, due to the low susceptibility of *Candida parapsilosis* and the development of secondary resistance by *Candida glabrata* reflected in the observed high rate of breakthrough infections. As previously reported [39,49], we could confirm IAC (67%) and candidemia (48%) as the main infection sites of invasive candidiasis in our population, with all cases of isolated candidemia being associated with the indwelling catheters.

Invasive aspergillosis (caused by *Aspergillus fumigatus*) occurred in 2% of our OLT recipients, despite an ongoing antifungal prophylaxis with clinical activity against *Aspergillus* spp. Even though OLT recipients are often predisposed to dissemination [50], none of our patients showed an extrapulmonary manifestation, most likely due to early-occurring death. While 80% of patients with IA died within the first 90 days (with IA as the primary cause of death), the mortality rate of IC was 24% (with IC as the primary cause of death in 5%), all of them caused by non-albicans species.

Despite the observed trend towards a rather later onset of IA (more than 90 days after OLT in the literature [51,52]), we observed most of the *Aspergillus* spp. infections emerging earlier, within a median of 36 (9–78) days. In comparison, invasive candidiasis was diagnosed quite a bit earlier, on average within 13 (3–44) days.

As already reported for the influenza and COVID-19-associated pulmonary aspergillosis, we found IA highly related to virus infections, which might partly explain their rather early occurrence in our cohort [53]. The majority of the described IA (60%) were associated with a preceding or concomitant virus disease (influenza A, CMV). In about a quarter of patients, an active CMV infection was present at the time of their death and half of them were associated with an IFI, while one patient died from tuberculosis reactivation. Moreover, half of the ongoing CMV infections at the time of death were external, or de novo infections of seronegative recipients, with half of the reactivations in seropositive recipients. As all donors have been tested seronegative before the OLT, no routine antiviral prophylaxis was performed.

Interestingly, 78% of the IC infections in deceased patients within the first year occurred as a breakthrough IFI during echinocandin prophylaxis. These infections were based upon the intrinsically reduced echinocandin susceptibility of non-albicans *Candida* spp., as well as upon a secondary echinocandin resistance of *C. glabrata* peritonitis after long-lasting echinocandin exposure. Another case of *C. orthopsilosis* also developed a secondary resistance to fluconazole during the treatment.

Although respiratory and fungal infections were more likely the cause of early mortality, the surgical site and soft tissue infections caused by MDR (*Enterobacteriaceae*, non-fermenting Gram-negative bacilli, and *Enterococcus faecium*) emerged as a major cause in the further postoperative course, reflecting complex surgical cases with a prolonged ICU stay and a coincidence of multiple postoperative complications.

Out of the 4% of patients who were transplanted with the high-urgent prioritization for primary acute liver failure, we found a 90-day survival rate of only 56%. This was significantly below the recently published data of the Eurotransplant region (80%) [54]. Finally, all other patients died from pneumonia-related sepsis (pulmonary aspergillosis, reactivation of tuberculosis, MDR pseudomonas) with coincident IC.

### 4.2. Risk Factors for Early Postoperative Mortality

Our multivariate model identified IFI as an independent risk factor for short-term mortality (HR 4.6, 95% CI 1.1–18.8; *p* = 0.032), raising the question of whether perioperative antimycotic management is the most appropriate modifiable factor with a significant impact on mortality. This is of particular importance since invasive fungal diseases are increasing globally [55].

Moreover, we confirmed that IFI (with *Candida* spp. being the most frequent organism, followed by *Aspergillus* spp.) have the strongest impact on mortality after OLT. Most of the cases occurred during the early postoperative period, with IAC as the most common one. Risk factors for IFI have been described earlier [52,56,57,58,59], and recommendations exist on a targeted antimycotic prophylaxis in high-risk OLT recipients [58]. Despite a proven reduction of IFI occurrence and its attributed mortality, a reduction in all-cause mortality in the case of antimycotic prophylaxis use has still not been proved [60]. Moreover, the international recommendations on the concrete substance and the duration of prophylaxis are still lacking.

Considerations on the antifungal spectrum, killing pattern and efficacy, as well as drug-related toxicity, possible interactions, pharmacokinetic profiles, and tissue penetration, make antimycotic prophylaxis a complex and controversial issue. American guidelines recommend the use of fluconazole, respectively, liposomal amphotericin B [61], but the persistent rise of non-albicans *Candida* spp. being less susceptible to triazoles led to the increased use of echinocandins [41,43,52,62,63]. Recent reports of secondary echinocandin resistance under long-lasting exposure and concerns about pharmacokinetic/pharmacodynamics variability in critically ill patients (both leading to increased rates of b-IFI) raise doubts on their use in a prophylactic regimen [64,65].

Finally, worrisome trends of co-resistance to both azoles and echinocandins in *C. glabrata* isolates, as well as increasing occurrences of multi-resistant *C. auris* and amphotericin B resistant *Aspergillus terreus*, have recently been published [66,67,68], pointing out the urgent need for new therapeutic options. The immediate fungicidal activity of current treatments is often reduced by a delayed therapeutic response. Drug-related toxicity and the emergence of resistance consequently further limit the therapeutic success and contribute to poor outcomes, keeping in mind that liver transplant recipients often show a high frailty with limited tolerance to additional organ toxicity or drug interactions. Therefore, the WHO formally recognizing IFI as being of critical importance to human health is certainly one of the first steps towards giving this topic the necessary awareness, and presents a basis for urgently needed further research funding [69].

### 4.3. Limitations

This study has several limitations. Due to the retrospective nature of our work, a selection bias may occur. However, all consecutive OLT recipients meeting the inclusion criteria were included in the final analysis. The data on sepsis were retrospectively taken from medical records. A detailed revalidation of the underlying parameters for the sepsis diagnosis according to the recent guideline definitions was not possible, and a possible bias cannot be excluded [70]. In contrast, all fungal findings, as a main statement of this study, were checked with a four-eyes principle and the diagnosis of IFI was made according to the described criteria. We performed a sensitivity analysis which included sepsis in the multivariate model, and we provide the results in the Supplementary. Moreover, the selective use of NMP for marginal donor organs (e.g., donation after circulatory determination of death), high-risk recipients, or difficult operations limit the validity of the statement on the possible influence on clinical outcome and the safety in clinical use, since the comparison to the standard risk recipients is missing. One of the most important limitations of our work may be related to its nature, as the retrospective identification of risk factors may lead to an underestimation of their true incidence. However, due to a relatively detailed and exact electronic medical documentation, a liberal approach to diagnostic modalities, and post-mortem examinations, the underestimation should be rather small. Lastly, our study comprised a comparably large cohort of OLT recipients, but larger samples and further studies are needed to clarify risk factors for mortality and the possible prevention of poor outcomes after transplantation.

### 4.4. Future Perspectives

In addition to the medium-term development of new diagnostic methods and antifungal substances, modern concepts of organ preservation such as NMP already enable a more efficient anti-infective treatment by opening a new window for additional diagnostics and interventions. Molecular genetics and microbiological investigations can significantly expand sometimes sparsely available donor data, especially when the donor has shown no clinical signs of infection so far. Although the examination results are often only available during or after the transplantation, and therefore have no influence on the decision of organ acceptance, the treating physicians still may gain the time advantage in the case of donor-derived infections. In the event of an actual infection transmission, the pathogen and its susceptibility will be known earlier, and further therapy can be better guided.

## 5. Conclusions

In this retrospective study from a transplant referral center, we found a mortality of 8% within 90 days and 14% within one year. Within the first year after OLT, 62% of all deaths were infection related. The IFI-incidence under targeted echinocandin use was 12%, with a case-fatality rate of 35%. These frequently underestimated infections are potentially modifiable and independent risk factor for short-term mortality. Moreover, two-thirds of postoperative *Candida* infections originated from intra-abdominal sites, where echinocandins exhibit poor tissue penetration. The high level of non-albicans *Candida* species exacerbates further concerns about their prophylactic use. Furthermore, despite the use of mold-active prophylaxis, two percent of patients developed early IA (often associated with viral diseases), with a remarkable 80% mortality. Finally, our results may support early and calculated therapy with the potential for an improvement in patient outcomes, highlighting the importance of IFI in the context of OLT.

## Figures and Tables

**Table 1:** Demographic and clinical characteristics of included patients (*n* = 214).

Patient Characteristics		All Patients (*n* = 214)	Survivors (*n* = 197)	Non-Survivors (*n* = 17)	*p*-Value	Missing (*n*/Total)
Age (years)		57.3 ± 11.1	57.4 ± 11.0	55.9 ± 13.2	0.582	0/214
Male sex		163 (76.2)	152 (77.2)	11 (64.7)	0.346	0/214
Height (cm)		174.2 ± 8.5	174.4 ± 8.4	171.7 ± 9.9	0.209	0/214
Weight (kg)		81.4 ± 16.3	81.9 ± 16.1	75.4 ± 18.6	0.114	0/214
Body mass index (kg/m^2^)		26.8 ± 5.0	26.9 ± 4.9	25.4 ± 5.3	0.236	0/214
SAPS III score		44.8 ± 8.5	44.6 ± 8.6	47.3 ± 7.7	0.240	6/214
MELD score		13 (6–40)	13 (6–40)	13.5 (6–39)	0.846	10/214
Charlson comorbidity index		4 (0–10)	4 (0–10)	5.5 (1–9)	0.213	2/214
**Underlying Disease**					0.033	0/214
Acute liver failure		10 (4.7)	7 (3.6)	3 (17.6)	0.035	
Tumors		89 (41.6)	84 (42.6)	5 (29.4)	0.319	
	Hepatocellular carcinoma	82 (92.1)	77 (91.7)	5 (100.0)	0.604	
	Cholangiocellular carcinoma	3 (3.4)	3 (3.6)	0 (0.0)	1.000	
	Neuroendocrine tumor	3 (3.4)	3 (3.6)	0 (0.0)	1.000	
	Polycystic liver disease	1 (1.1)	1 (1.2)	0 (0.0)	1.000	
Cirrhosis		68 (31.8)	64 (32.5)	4 (23.5)	0.591	
	Alcoholic cirrhosis	51 (23.8)	49 (24.9)	2 (11.8)	0.372	
	Virus related cirrhosis	9 (4.2)	9 (4.6)	0 (0.0)	1.000	
	Autoimmune cirrhosis	8 (3.7)	6 (3.0)	2 (11.8)	0.125	
Cholestatic disease		15 (7.0)	14 (7.1)	1 (5.9)	1.000	
Nonalcoholic steatohepatitis		14 (6.5)	12 (6.1)	2 (11.8)	0.307	
Metabolic disease		10 (4.7)	9 (4.6)	1 (5.9)	0.571	
Budd–Chiari syndrome		6 (2.8)	6 (3.0)	0 (0.0)	1.000	
Other		2 (0.9)	1 (0.5)	1 (5.9)	0.153	

Abbreviations: SAPS III: simplified acute physiology score III; MELD: model of end stage liver disease.

**Table 2:** Procedural data of analyzed population (*n* = 214).

Operative Characteristics		All Patients (*n* = 214)	Survivors (*n* = 197)	Non-Survivors (*n* = 17)	*p*-Value	Missing (*n*/Total)
Operation duration (minutes)		355 (173–783)	353 (173–783)	363 (175–783)	0.435	0/214
Cold ischemia time (minutes)		435 (125–1199)	441 (125–1199)	413 (240–1040)	0.867	0/214
Intraoperative blood transfusion (mL)		2412 (0–32,740)	2358 (0–21,200)	3300 (455–32,740)	0.066	0/214
Type of graft						0/214
	Whole liver	208 (97.2)	192 (97.5)	16 (94.1)	0.395	
	Split liver	6 (2.8)	5 (2.5)	1 (5.9)		
Type of biliary anastomosis						0/214
	Duct-to-duct	199 (93.0)	185 (93.9)	14 (82.4)	0.104	
	Roux-y-choledochojejunostomy	15 (7.0)	12 (6.1)	3 (17.6)		
Type of venous anastomosis						0/214
	Retrocaval resection	205 (97.2)	190 (97.9)	15 (88.2)	0.076	
	Piggyback	6 (2.8)	4 (2.1)	2 (11.8)		
**Donation and preservation characteristics**						
Type of donation						0/214
	Standard criteria donation	46 (21.5)	42 (21.3)	4 (23.5)	0.765	
	Extended criteria donation	168 (78.5)	155 (78.7)	13 (76.5)		
Type of donor death						0/214
	DBD	196 (91.6)	182 (92.4)	14 (82.4)	0.160	
	DCD	18 (8.4)	15 (7.6)	3 (17.6)		
Preservation						0/214
	Static cold storage	144 (67.3)	135 (68.5)	9 (52.9)	0.191	
	Normothermic machine perfusion	70 (32.7)	62 (31.5)	8 (47.1)		
Allocation						
	Local	42 (20.2)	39 (20.4)	3 (17.6)		6/208
	Regional	109 (52.4)	102 (53.4)	7 (41.2)	0.435	
	National	57 (27.4)	50 (26.2)	7 (41.2)		

Intraoperative blood transfusion includes packed red blood cells and autotransfusion of intraoperatively salvaged blood. Abbreviations: SAPS III: simplified acute physiology score III; MELD: model of end stage liver disease; DBD: donation after brain death; DCD: donation after circulatory determination of death.

**Table 3:** Postoperative complications (*n* = 214).

Postoperative Complications		All Patients (*n* = 214)	Survivors (*n* = 197)	Non-Survivors (*n* = 17)	*p*-Value	Missing (*n*/Total)
Length of ICU stay (days)		5 (1–117)	2 (1–117)	6 (1–40)	0.211	1/214
Graft dysfunction						0/214
	Primary non-function	2 (1.1)	2 (1.2)	0 (0.0)	1.000	
	Early allograft dysfunction	56 (30.9)	50 (30.5)	6 (35.3)	0.784	
Immunologic complications						0/214
	Acute rejection	10 (5.8)	8 (5.1)	2 (11.8)	0.256	
	Graft-versus-host disease	4 (2.3)	2 (1.3)	2 (11.8)	0.049	
Vascular complications						0/214
	Hepatic artery thrombosis	7 (3.4)	6 (3.2)	1 (6.3)	0.442	
	Portal vein thrombosis	6 (3.6)	4 (2.5)	2 (18.2)	0.051	
	Hepatic vein thrombosis	9 (5.4)	8 (5.1)	1 (9.1)	0.465	
Bile stricture		4 (2.2)	2 (1.2)	2 (11.8)	0.043	0/214
Reoperation		74 (34.6)	63 (32.0)	11 (64.7)	0.014	0/214
	Bile leak	20 (10.8)	18 (10.7)	2 (11.8)	1.000	
	Hemorrhage	31 (16.8)	23 (13.7)	8 (47.1)	0.002	
	Re-transplantation	6 (3.0)	6 (2.8)	0 (0.0)	1.000	
	Other	8 (4.4)	7 (4.1)	1 (7.1)	0.478	
Acute kidney injury		94 (43.9)	82 (41.6)	12 (70.6)	0.039	0/214
Sepsis		14 (8.1)	4 (2.6)	10 (58.8)	<0.001	0/214
CMV viremia		61 (28.5)	52 (26.4)	9 (52.9)	0.027	0/214
Invasive fungal infection		26 (12.1)	17 (8.6)	9 (52.9)	<0.001	0/214

Abbreviations: ICU: intensive care unit; CMV: cytomegalovirus.

**Table 4:** Postoperative complications in non-survivors (*n* = 17).

Complication	Number of Patients
Myocardial infarction	2 (2)
Posterior reversible encephalopathy syndrome and central pontine myelinolysis	1
Cerebrovascular accident	2 (2)
Acute kidney failure	12
Pancreatitis	1
Gastrointestinal perforation	1
Primary non-function	0
Early allograft dysfunction	6
Acute cellular rejection	2
Graft-versus-host-disease	2
Hospital-acquired and ventilator-associated pneumonia	6 (2)
Surgical site infection	7 (2)
Catheter-associated urinary tract infection	1 (1)
Central line-related bloodstream infection	5
Complicated skin and soft tissue infection	1
Sepsis	10 (10)
Invasive fungal infection	8 (5)
CMV viremia	9
Reoperation	11
Bile leak	2
Hemorrhage	8
Hepatic artery thrombosis	2 (2)
Portal vein thrombosis	3
Hepatic vein thrombosis	1

Number of patients who died from the complications is given in parentheses. Abbreviations: CMV: cytomegalovirus.

**Table 5:** Co-infections in patients with invasive aspergillosis (*n* = 5).

Primary Infection	Co-Infections		
VAP (*A. fumigatus*)	IAC + BSI (*C. dubliensis*)	VAP (*Influenza*)	SSI (*Citrobacter/MDR*)
VAP (*A. fumigatus)*	IAC + BSI (*C. orthopsilosis*, *C. krusei*)	VAP (*Influenza*)	VAP (*E. coli/MDR*)
VAP (*A. fumigatus*)	UTI (*C. glabrata*)	-	-
VAP (*A. fumigatus*)	-	-	-
VAP (*A. fumigatus*)	VAP (*Fusarium* spp., *Penicillium* spp.)	VAP/Colitis (CMV)	-

Abbreviations: VAP: ventilator-associated pneumonia; IAC: intra-abdominal candidiasis; BSI: blood stream infection; UTI: urinary tract infection; SSI: surgical site infection; MDR: multi-drug resistant.

**Table 6:** Multivariate analysis of risk factors for 90-day mortality (*n* = 214).

Non-Dependent Variable		B-Coefficient	*p*-Value	HR	95% Confidence Interval	
					Lower	Upper
Underlying disease (reference category: malignancy and other tumors)						
	Alcoholic Cirrhosis	−0.694	0.478	0.50	0.07	3.40
	Virus related Cirrhosis	-	-	-	-	-
	Nonalcoholic Steatohepatitis	1.541	0.089	4.67	0.79	27.52
	Budd-Chiari Syndrome	-	-	-	-	-
	Acute Liver Failure	1.146	0.252	3.14	0.44	22.33
	Cholestatic Disease	0.098	0.932	1.10	0.12	10.54
	Autoimmune Cirrhosis	1.033	0.328	2.81	0.36	22.24
	Metabolic Disease	0.904	0.420	2.47	0.28	22.21
	Other	0.038	0.979	1.04	0.06	17.18
Invasive Fungal Infection		1.534	0.032	4.64	1.14	18.78
Roux-Y-Choledochojejunostomy		−0.133	0.807	0.88	0.30	2.56
Piggyback-Anastomosis		1.469	0.226	4.35	0.40	46.87
Relaparotomy		0.157	0.823	1.17	0.30	4.62
Postoperative dialysis		0.460	0.457	1.58	0.47	5.32
CMV viremia		0.407	0.495	1.50	0.47	4.84
Graft-versus-host disease		1.011	0.381	2.75	0.29	26.34
Bile stricture		0.926	0.354	2.53	0.36	17.89
Portal vein thrombosis		0.784	0.385	2.19	0.37	12.84

Abbreviations: IFI: invasive fungal infections; CMV: cytomegalovirus; HR: hazard ratio.

## Data Availability

The datasets used and analyzed during the current study will be made available from the corresponding author on reasonable request.

## Supplementary Materials

The following supporting information can be downloaded at: https://www.mdpi.com/article/10.3390/jof9060655/s1, Supplementary Table S1. STROBE Statement—Checklist of items that should be included in reports of cohort studies; Supplementary Table S2. Characteristics of patients with procedure-related deaths (*n* = 4); Procedure-Attributed Mortality; Supplementary Table S3. Risk factors for mortality within 90 days of liver transplantation: univariate cox regression analyses (*n* = 214); Supplementary Table S4. Risk factors for mortality within 90 days of liver transplantation: multivariate analysis with sepsis (*n* = 214).
