# Supplementary material for: Invasive Fungal Infections: The Early Killer after Liver Transplantation
Source: J Fungi (Basel). 2023 Jun 12;9(6):655. doi: 10.3390/jof9060655 (PMC10301215; doi:10.3390/jof9060655)
Supplement: Supplementary file 1 [file jof-09-00655-s001.zip › jof-2435538-supplementary.pdf]

Supplementary material

Contents:

Supplementary Table S1. STROBE Statement - Checklist of items that should be included in reports of cohort studies

Supplementary Table S2. Characteristics of patients with procedure-related deaths (n = 4).

Procedure-Attributed Mortality

Supplementary Table S3. Risk factors for mortality within 90 days from liver transplantation: univariate cox regression analyses (*n* = 214)

Supplementary Table S4. Risk factors for mortality within 90 days from liver transplantation: multivariate analysis with sepsis (*n* = 214)

Supplementary Table S1. STROBE Statement - Checklist of items that should be included in reports of cohort studies

No.	Item	Recommendation	Page
	**Title and abstract**		
1		(*a*) Indicate the study's design with a commonly used term in the title or the abstract	1
		(*b*) Provide in the abstract an informative and balanced summary of what was done and what was found	1
	**Introduction**		
2	Background/rationale	Explain the scientific background and rationale for the investigation being reported	1-2
3	Objectives	State specific objectives, including any prespecified hypotheses	2-3
	**Methods**		
4	Study design	Present key elements of study design early in the paper	2-5
5	Setting	Describe the setting, locations, and relevant dates, including periods of recruitment, exposure, follow-up, and data collection	2-5
6	Participants	(*a*) Give the eligibility criteria, and the sources and methods of selection of participants. Describe methods of follow-up	2-5
		(*b*) For matched studies, give matching criteria and number of exposed and unexposed	2-5
7	Variables	Clearly define all outcomes, exposures, predictors, potential confounders, and effect modifiers. Give diagnostic criteria, if applicable	2-5
8*	Data sources/ measurement	For each variable of interest, give sources of data and details of methods of assessment (measurement). Describe comparability of assessment methods if there is more than one group	*2-5*
9	Bias	Describe any efforts to address potential sources of bias	2-5
10	Study size	Explain how the study size was arrived at	-
11	Quantitative variables	Explain how quantitative variables were handled in the analyses. If applicable, describe which groupings were chosen and why	-
12	Statistical methods	(*a*) Describe all statistical methods, including those used to control for confounding	4-5
		(*b*) Describe any methods used to examine subgroups and interactions	4-5
		(*c*) Explain how missing data were addressed	-
		(*d*) If applicable, explain how loss to follow-up was addressed	-
		(*e*) Describe any sensitivity analyses	-
	**Results**		
13*	Participants	(a) Report numbers of individuals at each stage of study—eg numbers potentially eligible, examined for eligibility, confirmed eligible, included in the study, completing follow-up, and analysed	5-6
		(b) Give reasons for non-participation at each stage	5-6
		(c) Consider use of a flow diagram	5-6
14*	Descriptive data	(a) Give characteristics of study participants (eg demographic, clinical, social) and information on exposures and potential confounders	5-7
		(b) Indicate number of participants with missing data for each variable of interest	-
		(c) Summarise follow-up time (eg, average and total amount)	2-5
15*	Outcome data	Report numbers of outcome events or summary measures over time	2-5
16	Main results	(*a*) Give unadjusted estimates and, if applicable, confounder-adjusted estimates and their precision (eg, 95% confidence interval). Make clear which confounders were adjusted for and why they were included	5-11
		(*b*) Report category boundaries when continuous variables were categorized	5-11
		(*c*) If relevant, consider translating estimates of relative risk into absolute risk for a meaningful time period	
17	Other analyses	Report other analyses done—eg analyses of subgroups and interactions, and sensitivity analyses	9-11
	**Discussion**		
18	Key results	Summarise key results with reference to study objectives	12

19	Limitations	Discuss limitations of the study, taking into account sources of potential bias or imprecision. Discuss both direction and magnitude of any potential bias	15
20	Interpretation	Give a cautious overall interpretation of results considering objectives, limitations, multiplicity of analyses, results from similar studies, and other relevant evidence	12-16
21	Generalisability	Discuss the generalisability (external validity) of the study results	12-16
	**Other information**		
22	Funding	Give the source of funding and the role of the funders for the present study and, if applicable, for the original study on which the present article is based	16

*Give information separately for exposed and unexposed groups.

Supplementary Table S2. Characteristics of patients with procedure-related deaths (n = 4).

Sex	Age	BMI	Indication	Risk factors	MELD	Urgency	Day of death	Cause of Death	Complications	Comment
f	64	18.0	ALF (HHV-6)	HE III/IV°, INR >3.5, bilirubin >300 mmol/l	33	HU	1	Circulatory failure	-	CPR during hepatectomy (ROSC after 1 minute), circulatory failure during ischemia-reperfusion-syndrome with *mors in tabula*
m	63	32.9	Tumor (HCC)	Preexisting PVT, thromboembolic disease history (DVT/PE), metabolic syndrome (hypertension, obesity, dyslipidemia, NIDDM, COPD)	8	T	3	Hepatic necrosis	Hemorrhage, ACS, AKI	PV reconstruction by interpositional EIV graft replacement, frustrane anastomosis to the SMV, perihepatic packing and external biliary drainage, early repacking (POD 1), unpacking and duct-to-duct biliary reconstruction (POD 2)
m	69	22.0	Metabolic (AAT)	Preexisting PVT, coagulation disorder (prothrombin mutation)	12	T	2	Hepatic necrosis	Hemorrhage, early HAT, AKI	
f	69	25.4	Tumor (HCC)	Donor-related risk factors: HAV accessory left hepatic artery from the LGA, Michel´s type V	7	T	226	Ischemic Cholangiopathy	Early HAT (POD 22), AKI	End-to-end anastomosis of the recipient CHA and graft trunk, OLT duration 376 minutes, anastomosis duration: 38 min., anticoagulation with UFH for 24 hours.

Abbreviations: BMI: body mass index; f: female; m: male; ALF: acute liver failure; HHV-6: human herpesvirus-6; HCC: hepatocellular carcinoma; INR: international normalized ratio; AAT: alpha-1 antitrypsin deficiency; HE: hepatic encephalopathy; HAV: hepatic artery variant; LGA: left gastric artery; DVT: deep vein thrombosis; PE: pulmonary embolism; COPD: chronic obstructive pulmonary disease; PVT: portal vein thrombosis; HU: high-urgency; T: transplantable; HAT: hepatic artery thrombosis; ACS: abdominal compartment syndrome; AKI: acute kidney injury; CPR: cardiopulmonary resuscitation; ROSC: return of spontaneous circulation; CHA: common hepatic artery; PV: portal vein; EIV: external iliac vein; SMV: superior mesenteric vein; UFH: unfractionated heparin; POD: postoperative day; NIDDM: non-insulin dependent diabetes mellitus.

Procedure-Attributed Mortality

In total 4 patients died due to procedure-related complications. The first patient died due to a circulatory decompensation during the reperfusion period in the context of a preceding human herpesvirus-6 associated acute liver failure, which was postoperatively confirmed by PCR in the biopsy material of the explanted liver. The second and third patient died as a consequence of a preexisting postoperative portal vein thrombosis (PVT) with a complex surgical course and finally, one due to an early HAT, Supplementary Table 2. Moreover, one of these patients developed a late graft-failure (more than 90 days after OLT) due to an early HAT, which was diagnosed in computed tomographic arteriography (elevated transaminases, 22nd day after OLT). The patient was listed for re-transplantation 75 days after OLT, but deceased on the waiting list on the 226th day after OLT. All procedural complications were related to known perioperative risk factors.

Most of the procedure-related deaths were associated with vascular complications, due to complex surgical techniques in patients with preexisting PVT or vascular variations with perioperative massive transfusion. Most of them coincided with recipient-related risk factors (e.g., metabolic syndrome, a history of thromboembolic events, thrombophilia, etc.).

Preexisting PVT is a common problem in patients with cirrhosis, with the need for complex clot removal or vascular reconstructions, having negative impact on early postoperative patient and graft survival [1-4]. Likewise, the occurrence of an early HAT can be associated with a higher rate of mortality and graft lost [5]. In our cohort, we found an early HAT incidence of 3.3%, which is comparable to the published data [6]. Most of these patients (71%) could be re-transplanted (60% of these successfully), while one patient was successfully treated by percutaneous transluminal angioplasty. Consistent with previously reported evidence, the 90-day mortality of early HAT was 29%, despite the performed treatments [6].

The majority of early deaths were associated with a coincidence of multiple postoperative complications including presence of acute kidney injury (44%). Acute kidney injury is a common postoperative OLT complication with documented influence on long-term morbidity and with rates exceeding 50% [7,8].

References:

1. Rana, A.; Hardy, M.A.; Halazun, K.J.; Woodland, D.C.; Ratner, L.E.; Samstein, B.; Guarrera, J.V.; Brown, R.S.; Emond, J.C. Survival Outcomes Following Liver Transplantation (SOFT) Score: A Novel Method to Predict Patient Survival following Liver Transplantation. *Am. J. Transpl.* **2008**, *8*, 2537–2546. https://doi.org/10.1111/J.1600-6143.2008.02400.X.
2. Ponziani, F.R.; Zocco, M.A.; Senzolo, M.; Pompili, M.; Gasbarrini, A.; Avolio, A.W. Portal Vein Thrombosis and Liver Transplantation: Implications for Waiting List Period, Surgical Approach, Early and Late Follow-Up. *Transpl. Rev.* **2014**, *28*, 92–101. https://doi.org/10.1016/J.TRRE.2014.01.003.
3. Zanetto, A.; Rodriguez-Kastro, K.I.; Germani, G.; Ferrarese, A.; Cillo, U.; Burra, P.; Senzolo, M. Mortality in Liver Transplant Recipients with Portal Vein Thrombosis—An Updated Meta-Analysis. *Transpl. Int.* **2018**, *31*, 1318–1329. https://doi.org/10.1111/TRI.13353.
4. Ghabril, M.; Agarwal, S.; Lacerda, M.; Chalasani, N.; Kwo, P.; Joseph Tector, A. Portal Vein Thrombosis Is a Risk Factor for Poor Early Outcomes after Liver Transplantation: Analysis of Risk Factors and Outcomes for Portal Vein Thrombosis in Waitlisted Patients. *Transplantation* **2016**, *100*, 126–133. https://doi.org/10.1097/TP.0000000000000785.
5. Valente, J.F.; Alonso, M.H.; Weber, F.L.; Hanto, D.W. Late Hepatic Artery Thrombosis in Liver Allograft Recipients Is Associated with Intrahepatic Biliary Necrosis. *Transplantation* **1996**, *61*, 61–65. https://doi.org/10.1097/00007890-199601150-00013.
6. Bekker, J.; Ploem, S.; De Jong, K.P. Early Hepatic Artery Thrombosis after Liver Transplantation: A Systematic Review of the Incidence, Outcome and Risk Factors. *Am. J. Transpl.* **2009**, *9*, 746–757. https://doi.org/10.1111/J.1600-6143.2008.02541.X.
7. Hilmi, I.A.; Damian, D.; Al-Khafaji, A.; Planinsic, R.; Boucek, C.; Sakai, T.; Chang, C.C.H.; Kellum, J.A. Acute Kidney Injury Following Orthotopic Liver Transplantation: Incidence, Risk Factors, and Effects on Patient and Graft Outcomes. *Br. J. Anaesth.* **2015**, *114*, 919–926. https://doi.org/10.1093/BJA/AEU556.
8. Durand, F.; Francoz, C.; Asrani, S.K.; Khemichian, S.; Pham, T.A.; Sung, R.S.; Genyk, Y.S.; Nadim, M.K. Acute Kidney Injury after Liver Transplantation. *Transplantation* **2018**, *102*, 1636–1649. https://doi.org/10.1097/TP.0000000000002305.

Supplementary Table S3. Risk factors for mortality within 90 days from liver transplantation: univariate cox regression analyses (n = 214)

Nondependent variable	B-coefficient	P-value	HR	95% confidence interval lower	upper
Age (years)	-0.010	0.610	0.99	0.95	1.03
Sex (male)	0.576	0.256	1.78	0.66	4.81
Height (cm)	-0.038	0.190	0.96	0.91	1.02
Weight (kg)	-0.025	0.113	0.98	0.95	1.01
Body mass index (kg/m2)	-0.062	0.243	0.94	0.85	1.04
SAPS III score	0.038	0.237	1.04	0.98	1.11
MELD score	0.032	0.243	1.03	0.98	1.09
Charlson comorbidity index	0.127	0.224	1.14	0.93	1.39
Intensive care length of stay (days)	0.011	0.307	1.01	0.99	1.03
Cold ischemia time (minutes)	0.001	0.335	1.00	1.00	1.00
Warm ischemia time (minutes)	0.933	0.371	1.00	1.00	1.01
Surgery duration (minutes)	0.001	0.114	1.00	1.00	1.00
Metra time	0.001	0.141	1.00	1.00	1.00
Invasive fungal infection	2.157	<0.001	8.64	3.33	22.43
Breakthrough IFI	1.803	0.002	6.07	1.98	18.65
Transplantation year	0.054	0.806	1.06	0.69	1.63
Weekend	-0.032	0.953	0.97	0.34	2.75
Weekday	0.074	0.544	1.08	0.85	1.37
Age gap between donor and recipient (years)	-0.016	0.229	0.98	0.96	1.01
Age gap (10 years)	0.785	0.170	2.19	0.72	6.72
Age gap (20 years)	-0.421	0.508	0.66	0.19	2.28
**Underlying disease malignancy and other tumours (reference category)**					
Alcoholic liver disease	-0.386	0.645	0.68	0.13	3.51
Virus related	-	-	-	-	-
Non-alcoholic fatty liver disease	0.986	0.239	2.68	0.52	13.82
Budd-Chiari syndrome	-	-	-	-	-
Acute liver failure	1.785	0.015	5.96	1.42	24.97
Cholestatic	0.183	0.867	1.20	0.14	10.28
Autoimmune hepatitis	1.566	0.061	4.79	.93	24.70
Metabolic liver disease	0.630	0.565	1.88	0.22	16.08
Other	2.220	0.043	9.21	1.07	78.94
Prehospitalization	0.773	0.177	2.17	0.71	6.64
Fungal colonization at baseline	1.084	0.089	2.96	0.85	10.29
Pretransplant serum creatinine >2 mg/dl	0.943	0.210	2.57	0.59	11.24
Early allograft dysfunction	0.435	0.391	1.55	0.57	4.18
Primary non-function	-3.015	-	-	-	-
Acute cellular rejection	0.967	0.199	2.63	0.60	11.50
Graft-versus-host disease	2.040	0.007	7.69	1.76	33.70
Sepsis	3.272	<0.001	26.36	9.92	70.08
Bile leak	0.242	0.748	1.27	0.29	5.57
Bile stricture	1.971	0.009	7.18	1.64	31.42
Hepatic artery thrombosis	0.705	0.494	2.02	0.27	15.26
Hepatic vein thrombosis	0.377	0.714	1.46	0.19	10.99

Portal vein thrombosis	1.600	0.034	4.96	1.13	21.68
Hemorrhage	1.750	<0.001	5.76	2.22	14.93
Piggyback operative technique	1.654	0.028	5.23	1.20	22.88
Preservation (static cold storage)	0.602	0.215	1.83	0.70	4.73
Choledochojejunostomy, primary	1.075	0.091	2.93	0.84	10.20
Blood transfusion	0.314	0.188	1.37	0.86	2.18
Massive transfusion (more than 10 units)	0.361	0.457	1.46	0.55	3.72
Split liver transplantation	0.755	0.464	2.13	0.28	16.04
Donor derived infection	-	-	-	-	-
High-urgency transplantation	1.711	0.007	5.53	1.59	19.27
Extended criteria donation	-0.118	0.837	0.89	0.29	2.73
Donation after circulatory determination of death	0.869	0.172	2.38	0.69	8.30
Bile leak	0.473	0.408	1.61	0.52	4.92
Reoperation	1.266	0.013	3.55	1.31	9.59
Early re-transplantation	-	-	-	-	-
Posttransplant dialysis	1.148	0.031	3.15	1.11	8.95
CMV viremia	1.054	0.030	2.87	1.11	7.44
**Donor related factors**					
Age (years)	0.016	0.325	1.02	0.99	1.05
Sex (male)	0.252	0.604	1.29	0.50	3.33
Height (cm)	-0.035	0.182	0.97	0.92	1.02
Weight (kg)	-0.020	0.237	0.98	0.95	1.01
Body mass index (kg/m2)	-0.022	0.699	0.98	0.88	1.09
HBV positive	-	-	-	-	-
HCV positive	-	-	-	-	-
Prolonged hypotensive period	-0.486	0.445	0.62	0.18	2.14
Hypernatremia (>155 mmol/l)	-0.555	0.604	0.57	0.07	4.67
Nosocomial infection	0.360	0.736	1.43	0.18	11.65
Ventilation duration (days)	-0.190	0.740	0.83	0.27	2.54

Abbreviations: IFI, invasive fungal infections; b-IFI, breakthrough IFI; SAPS III, simplified acute physiology score III; MELD, Model for end-stage liver disease score; CMV, cytomegalovirus; BMI, body mass index; HBV, hepatitis B virus; HCV, hepatitis C virus; HR, hazard ratio.

Supplementary Table S4. Risk factors for mortality within 90 days from liver transplantation: multivariate analysis with sepsis (n = 214)

Nondependent variable	B-coefficient	P-value	HR	95% confidence interval	
				lower	upper
Underlying disease (reference category: malignancy and other tumours)					
Alcoholic cirrhosis	-1.828	0.161	0.16	0.01	2.07
Virus related cirrhosis	-	-	-	-	-
Nonalcoholic steatohepatitis	1.646	0.106	5.18	0.71	38.09
Budd-Chiari syndrome	-	-	-	-	-
Acute liver failure	0.522	0.633	1.69	0.19	14.37
Cholestatic disease	0.578	0.623	1.78	0.18	17.88
Autoimmune cirrhosis	1.039	0.302	2.83	0.39	20.36
Metabolic disease	1.453	0.219	4.28	0.42	43.33
Other	-2.182	0.194	0.11	0.01	3.04
Sepsis	3.844	<0.001	46.71	7.86	277.65
Roux-Y-choledochojejunostomy	0.381	0.460	1.46	0.53	4.02
Piggyback anastomosis	2.296	0.078	9.93	0.78	127.12
Relaparotomy	0.053	0.941	1.05	0.26	4.29
Postoperative dialysis	-0.431	0.579	0.65	0.14	2.98
CMV viremia	-0.008	0.991	0.99	0.25	4.00
Graft-versus-host disease	3.268	0.026	26.25	1.48	466.70
Bile stricture	-0.756	0.465	0.47	0.06	3.58
Portal vein thrombosis	-0.784	0.554	0.46	0.03	6.11

Variables excluded from model (multicollinearity): hemorrhage, high-urgency, breakthrough IFI, fungal colonization at baseline, and IFI. Abbreviations: IFI, invasive fungal infections; HR, hazard ratio.
